# Regulation of craving for real-time fMRI neurofeedback based on individual classification

**DOI:** 10.1098/rstb.2023.0094

**Published:** 2024-10-21

**Authors:** Dong-Youl Kim, Jonathan Lisinski, Matthew Caton, Brooks Casas, Stephen LaConte, Pearl H. Chiu

**Affiliations:** ^1^ Fralin Biomedical Research Institute at VTC, Virginia Tech, Roanoke, VA, USA; ^2^ Department of Psychology, Virginia Tech, Blacksburg, VA, USA; ^3^ Department of Biomedical Engineering and Mechanics, Virginia Tech, Blacksburg, VA, USA

**Keywords:** classifier optimization, individual classification, neurofeedback, real-time fMRI, smoking craving, support vector machine

## Abstract

In previous real-time functional magnetic resonance imaging neurofeedback (rtfMRI-NF) studies on smoking craving, the focus has been on within-region activity or between-region connectivity, neglecting the potential predictive utility of broader network activity. Moreover, there is debate over the use and relative predictive power of individual-specific and group-level classifiers. This study aims to further advance rtfMRI-NF for substance use disorders by using whole-brain rtfMRI-NF to assess smoking craving-related brain patterns, evaluate the performance of group-level or individual-level classification (*n* = 31) and evaluate the performance of an optimized classifier across repeated NF runs. Using real-time individual-level classifiers derived from whole-brain support vector machines, we found that classification accuracy between crave and no-crave conditions and between repeated NF runs increased across repeated runs at both individual and group levels. In addition, individual-level accuracy was significantly greater than group-level accuracy, highlighting the potential increased utility of an individually trained whole-brain classifier for volitional control over brain patterns to regulate smoking craving. This study provides evidence supporting the feasibility of using whole-brain rtfMRI-NF to modulate smoking craving-related brain responses and the potential for learning individual strategies through optimization across repeated feedback runs.

This article is part of the theme issue ‘Neurofeedback: new territories and neurocognitive mechanisms of endogenous neuromodulation’.

## 1. Introduction

Despite smoking being a leading risk factor for early death, more than one in five adults in the United States smoke tobacco cigarettes and nearly one in four adults smoke tobacco cigarettes worldwide [1–3]. To help improve health outcomes, intensive efforts have been made to develop and augment interventions that support individuals who are trying to quit smoking. Growing research suggests that craving plays a mechanistic role in smoking maintenance and that learning to regulate craving may reduce relapse [4]. Giving smokers insight into their craving through real-time functional magnetic resonance imaging (rtfMRI) neurofeedback (NF) is promising for facilitating regulatory control of craving.

Many studies have provided evidence for the feasibility of voluntarily modulating functional processes in the human brain using rtfMRI-NF approaches [5–19]. In the past decade, there has been increased interest in the potential application of rtfMRI-NF techniques for neurological and psychiatric disorders, including substance use disorders. Specifically, rtfMRI-NF has been modestly successful for regulating smoking cravings in tobacco cigarette smokers [11,20–24]. In early studies [20–22], the NF signal was generated through the targeting of regions of interest (ROIs) such as the medial frontal cortex, a region implicated in cigarette cravings triggered by visual cues associated with smoking. More recently, Karch and colleagues investigated the efficacy of modulating haemodynamic activity within ROIs of the anterior cingulate cortex (ACC), dorsolateral prefrontal cortex and insula for reducing smoking craving using traditional activity-based rtfMRI-NF approaches [23,24].

While rtfMRI-NF methods have most often been based on feedback from activity within ROIs, the modulation of activity has been shown to extend beyond the targeted ROIs to connectivity patterns across multiple brain regions [10,25–28]. Rota and colleagues, through examination of the regulation of activity in regions implicated in prosody processing, found that rtfMRI-NF training modulated functional and effective connectivity throughout the brain [27]. Accordingly, machine learning techniques such as support vector machines (SVMs) have been used on network-based regions and whole brain to extract NF information rather than solely on activity levels within ROIs [29–35]. For example, Fede and colleagues compared the performance of different types of NF across activity in multiple regions using both intermittent feedback of SVM and continuous feedback of SVM, finding superior performance for modulating alcohol dependence-related processes when continuous feedback was used relative to other conditions [35]. This classifier-based approach, called decoded NF, has been repeatedly suggested as having potential for improving the modulation of brain states [6,36,37].

While recent research groups have applied machine learning techniques to whole-brain or ROI activity to extract feedback information, no study to our knowledge has compared the performance of NF using group-level versus individual-level classifiers, despite data demonstrating that individualized NF learning strategies are effective for modulating brain function and corresponding cognitive processes [13,32,38–41]. Thus, in this study, we investigated the performance of classifiers derived from group-level information or individual-level information. In addition, based on a recent classifier-based NF study that updated classifiers across repeated NF runs and reported significant improvement in classification accuracy [42], we used a classifier-based NF approach adopting a pre-defined and updated model for optimizing NF information across real-time NF runs. We hypothesized that a rtfMRI-NF method with individually updated classifiers across repeated training runs would result in enhanced modulation of neural information in brain areas implicated in cigarette cravings.

To examine this possibility, we investigated whether updating individual classifiers across repeated NF runs affected the classification performance of subsequent runs and thus could provide improved NF information to the participant for modulating smoking craving. More specifically, we adopted SVMs to classify whole-brain activation associated with instructions to ‘crave’ or ‘don’t crave’ while participants viewed smoking-related images and evaluated the performance of individual- and group-level classifiers to discriminate between repeated runs or between ‘crave’ and ‘don’t crave’ conditions.

## 2. Material and methods

### (a) Participants

The research protocol was approved by Virginia Tech’s Institutional Review Board (IRB). All subjects completed the informed consent process prior to participation and were compensated in accordance with IRB policy. Eighty-seven adults (male = 43, age = 32.4 ± 11.5 years) were screened over the course of 1 year.

The inclusion criteria were as follows: aged 18–55, right-handed, vision corrected to be able to see the computer display clearly with or without eyeglasses, fluency in English, smoking at least five cigarettes per day, smoking cigarettes for at least the past year, willing to abstain from cigarette use for at least 12 h prior to imaging and not trying to quit smoking. Exclusion criteria included left-handed, claustrophobia, Diagnostic and Statistical Manual of Mental Disorders-IV Axis I or II [43] current diagnosis exclusive of nicotine dependence, current pregnancy, contraindications to MRI (pacemaker, aneurysm clips, neurostimulators, cochlear implants, metal in eyes, steel worker or other implants), active neurologic disorder, history of alcohol or drug dependence (excluding nicotine) and history of head injuries resulting in loss of consciousness for more than 10 min. Inclusion and exclusion criteria were assessed via telephone and confirmed during the first visit. Smoking status was assessed at the first visit via self-report and breath carbon monoxide (CO; Smokerlyzer, Bedfont Scientific, Ltd., Rochester, UK).

Thirty-seven individuals met the full entrance criteria and participated in the study. Six of these participants were excluded from the analysis due to head movement (absolute translation parameter at any direction > 2 mm). The screening procedure for recruiting participants is illustrated in electronic supplementary material, figure S1. Table 1 summarizes the demographic and behavioural data obtained from the final sample of participants in the analysis (*n* = 31).

**Table 1.** Summary of demographic and smoking-related variables.^a^ FTND, Fagerström test of nicotine dependence [44]; SJWS, Shiffman–Jarvik withdrawal scale [45].

	*n =* ***31***
age (years)	32.97 (11.54)
gender (#male/female)	15/16
cigarettes/day	15.03 (7.84)
cigarette use form	13.65 (6.87)
FTND	4.61 (2.28)
SJWS total score	100.87 (24.08)
SJWS smoking items	41.74 (6.87)
CO level in the lungs (ppm)	14.45 (8.05)^b^
CO level in the blood (%Hb)	2.94 (1.29)^b^

^a^ The data are presented as the mean values (and standard deviation).

^b^ *n* = 29 due to missing two subjects.

### (b) Experimental set-up

**Figure 1.**
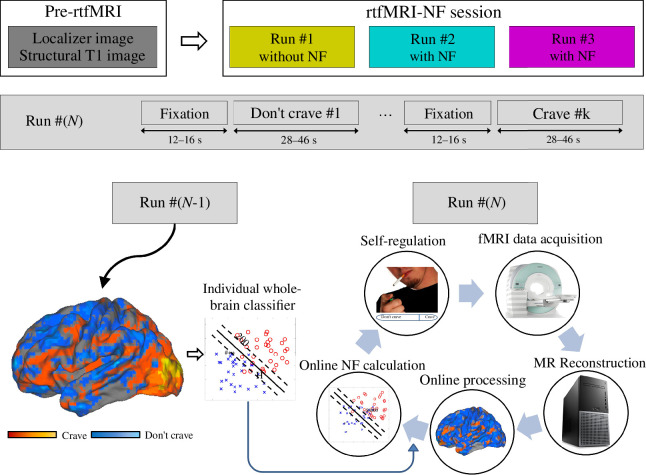
Experimental set-up of rtfMRI-NF. (Top) Flow diagram showing the protocol and task paradigms. (Middle) Block-based design in each rtfMRI-NF run. (Bottom) Information on the NF phase of each rtfMRI-NF run and how to apply the classifier from prior run to following run.

As illustrated in figure 1, each participant underwent a localizer and structural run, followed by three rtfMRI runs. The first rtfMRI run without NF lasted 684 s (excluding the initial 6 s) and consisted of pseudo-random alternating and counterbalanced periods of instructions to ‘crave’ (*n* = 6; jittered 34–46 s) or ‘don’t crave’ (*n* = 6; jittered 34–46 s) while viewing smoking-related images [46]. The smoking-related images were presented in a randomized order across all the blocks and runs, and the craving intensity ratings [46] across images did not differ between pairs of runs (i.e. *p* = 0.92, 0.89 and 0.97 for the first versus second runs, second versus third runs and first versus third runs, respectively, from two-sample *t*‐test). These instructed periods were interleaved by fixation periods (*n* = 13; jittered 12–16 s) that served as ‘baseline’ comparisons. The instruction given to the participant was to try to ‘keep fixating on the crosshair, remain at rest with your eyes open, and not think of any mental strategies’. The second and third rtfMRI runs with NF lasted 768 s (excluding the initial 6 s) and similarly consisted of pseudo-random alternating and counterbalanced blocks of ‘crave’ (*n* = 7; jittered 28–46 s) and ‘don’t crave’ conditions (*n* = 7; jittered 28–46 s) interleaved by fixations (*n* = 15; jittered 12–16 s). Participants were provided with example strategies to use such as imagining ‘how a cigarette looks, smells, feels, and how it would taste right now’ for the ‘crave’ condition and ‘the bad tastes or smells or feeling unhealthy’ for the ‘don’t crave’ condition. This allowed participants to freely choose their own strategies to modulate their craving status. Additionally, participants were informed that brain patterns would be related to craving states but were not given details on how the NF information was estimated and reflected. The NF was delivered to participants through a moveable slider below the images and no feedback was provided during the first ‘crave’ and ‘don’t crave’ blocks. The first blocks during the NF runs were not used in further analysis.

### (c) Imaging parameters

Blood oxygen level-dependent (BOLD) fMRI data were acquired using a 3 T Siemens Tim-Trio scanner with a 12-channel head coil (Erlangen, Germany). A standard gradient-echo echo-planar-imaging (EPI) pulse sequence was applied to measure BOLD intensity associated with brain activity levels across the whole brain. The EPI parameters of the rtfMRI-NF run were time of repetition (TR) = 2000 ms, time of echo (TE) = 30 ms, field of view (FoV) = 220 × 220 mm^2^, matrix size = 64 × 64, voxel size = 3.4 × 3.4 × 4.0 mm^3^, flip angle (FA) = 90°, and 33 interleaved slices. The T1-weighted image was acquired with the following parameters: Generalized Autocalibrating Partial Parallel Acquisition—TR = 2600 ms, TE = 3.02  ms, FoV = 256 × 256  mm^2^, voxel size = 1 × 1 × 1  mm^3^, FA = 8°, 192 sagittal slices with no gap.

### (d) Neurofeedback information estimation in real time

The rtfMRI-NF system was based on previous studies (detailed in supplementary materials; [6,34]). Two preliminary runs in each session were required to enable rtfMRI-NF. The first run required about 8 s of data acquisition to collect four fMRI volumes. This short run was used to define the slice prescriptions of all subsequent runs and produced a brain mask generated by AFNI’s 3dAutomask command. To calculate the NF signals, the whole-brain BOLD signals from the prior run were preprocessed with slice timing correction and motion correction using AFNI. The preprocessed BOLD time series without the first two volumes of every block were entered to make a predictive model to classify the ‘crave’ and ‘don’t crave’ conditions using 3dsvm (three-dimensional SVM with a linear kernel; parameter of SVM, C = 200 demonstrated in a previous study [6]) using AFNI. In the following run, the preprocessed BOLD time series with the same options were used to classify the two conditions (‘crave’ or ‘don’t crave’) in a volume-wise manner. Therefore, 3dsvm used transmission control protocol/internet protocol communication to transmit unthresholded classifier output to control a slider-bar interface implemented in Vision Egg [47] (example in electronic supplementary material, video S1). Positive and negative classifier distances from the hyperplane were defined as ‘crave’ and ‘don’t crave’ classified labels, respectively. A running linear trend of the distance (*d*) to the hyperplane was calculated to correct for temporal drifts and an offset of the mean value of the classifier. The absolute distance was discretized into four bins (|*d*| < 0.2, |*d*| < 0.4, |*d*| < 0.6, |*d*| < 0.8 and |*d*| ≥ 0.8) to avoid overly small movements. The slider bar integrated the feedback signal within a block and the step size for each volume was set based on the block length and, therefore, ideal classifier performance was at a bin of |*d*| ≥ 0.8.

### (e) Offline analysis: preparation for classification

The acquired EPI volumes from each rtfMRI-NF run were preprocessed using a series of steps including head motion correction, spatial normalization and spatial smoothing using an 8 mm isotropic full-width at half-maximum Gaussian kernel as implemented in SPM12. The resulting BOLD fMRI time-series data were further processed using temporal smoothing across three volume periods (3 s) followed by temporal detrending to significantly reduce low-frequency linear drift noise [48–50].

The BOLD time series of the voxels on the whole brain were converted to the percentage BOLD signal changes (PSC) by adjusting a baseline BOLD intensity, which was defined as the average below zero of the convolved time series with haemodynamic response function (HRF). This baseline was assumed to reflect the rest taken by the participants during the fixation blocks. The estimated PSC was used for the samples of within-subject classification.

For group-level classification, the general linear model (GLM) was applied to the preprocessed BOLD fMRI data. Onset timing and duration of each ‘crave’ and ‘don’t crave’ block were used to model a reference HRF of each block using SPM12. Then, the block-wise regressors across all the conditions were defined as the result of convolved signals between the HRF and block-wise information of onset and duration. For each participant, neuronal activation patterns were defined by the beta values of the GLM across voxels within the whole brain.

### (f) Offline analysis: individual- and group-level classifications

For individual-level classification to compare pairs of repeated NF runs, the PSC were normalized between 0 and 1 across volumes (i.e. # of volumes = 144 for each of the ‘crave’ and ‘don’t crave’ conditions of each of the three runs, six blocks for each run, the first block excluded for the second and third runs) in the training data (i.e. normalization with a range from 0 to 1 across the number of voxels × the number of volumes used), and the scaling factors for this normalization were applied to the validation and test data. There were nine contrasts in total: (i) the first run versus the second run, (ii) the first run versus the third run and/or (iii) the second run versus the third run in (i) ‘crave’ only, (ii) ‘don’t crave’ only and/or (iii) collapsing across ‘crave’ and ‘don’t crave’ conditions. For the contrast to compare pairs of repeated runs, an explicit mask excluding the visual regions such as the bilateral calcarine, cuneus, lingual gyrus and superior, middle and inferior occipital lobes from the Automated Anatomical Labeling [51] and Brodmann area 17 [52] was used. A ν-SVM classifier with a linear kernel was used as implemented in the LIBSVM software toolbox (https:\\www.csie.ntu.edu.tw/~cjlin/libsvm). The latent parameter ν was optimized via a grid search using uniformly distributed candidate values (from 0.1 to 0.8, with an interval of 0.1) [53]. The nested cross-validation classification test [54–56] was repeated 20 times using 20 randomly shuffled training, validation and test sets, and average classification accuracies are reported. The volume-wise PSC were divided into 10 folds: eight folds (*n* = 114) for training a classifier, one fold (*n* = 15) for validation and one remaining fold (*n* = 15) for testing.

For group-level classification of pairs of repeated NF runs, the voxel-wise beta values were normalized between 0 and 1 across subjects in the training data (i.e. normalization with a range from 0 to 1 across the number of voxels × the number of subjects used), and the scaling factors for this normalization were applied to the validation and test data. The same framework as used for individual classification was adopted including nested cross-validation, with a different number of permuted tests such as 100 times using 100 randomly shuffled training, validation and test sets. Given the relatively small number of samples for group-level classification, more permuted sets were adopted for group-level classification than individual-level classification to increase reliability. The subject-wise beta maps were divided into 10 folds: eight folds (*n* = 24) for training a classifier, one fold (*n* = 3) for validation and one remaining fold (*n* = 4) for testing.

To compare the classification accuracies between the two contrasts from group classification, a *post-hoc* two-sample *t*‐test was conducted. A linear regression model was performed to compare the accuracies across the three contrasts. For comparison of accuracies between the two contrasts from individual classification, a paired *t*‐test was adopted. Bonferroni correction was applied to the *t*‐test (*n* = 100 and 31 for group and individual classification, respectively) and the regression model with a number of samples used at that test.

To compare ‘crave’ and ‘don’t crave’ conditions within each NF run, for both individual and group classification, the input data were prepared in the same manner as those for comparing pairs of NF runs, described above. The same cross-validation framework was applied to optimize the latent parameter and calculate individual and group classification accuracy. In addition, the classifier from each run, in both group and individual classification, was tested on the other runs to evaluate each classifier’s predictive performance in the other runs. For comparison between the online and offline analyses, additional analysis in the group and individual classifications was conducted using c-SVM with the same scheme adopted in the case of ν-SVM. To compare the classification accuracies between and/or across the contrasts, the *post-hoc* analysis was performed in the same manner as described above.

To assess potential effects of motion on classification performance between the ‘crave’ and ‘don’t’ crave’ conditions, framewise displacement (FD) was measured during the volumes across blocks for the two conditions [57]. Mean FD values were averaged across the volumes for each of the two conditions. A paired *t*‐test was employed to compare motion parameters between the two conditions and Bonferroni correction was applied to the *t*‐test (*n* = 31). In addition, regression analysis between mean FD values and individual accuracy in classifying the ‘crave’ and ‘don’t crave’ conditions was performed to examine the effect of motion differences between the two conditions on individual classification performance with Bonferroni correction.

### (g) Offline analysis: identification of brain regions

The whole-brain weight features from the 31 participants for individual classification between ‘crave’ and ‘don’t crave’ conditions or between pairs of runs were normalized via pseudo *z*-scoring on the whole brain for each subject and were subjected to a one-sample *t*‐test for group inference. To compare the brain features across repeated runs, one-way analysis of variance (ANOVA) was conducted with repeated runs as a within-subject independent variable. The statistical significance of group inference was estimated from 10 000 tests performed using randomly permuted sets with randomly assigned class labels for the test sets [56,58,59].

### (h) Offline analysis: regression analysis using behavioural measurements and weight feature value

The ROIs identified from group inference via ANOVA were further used for regression analysis. Simple linear regressions were performed to estimate associations between the brain features as averaged weight feature values within each ROI and the smoking-related behavioural measurements such as cigarettes per day, cigarette use form, a Fagerström test of nicotine Ddpendence score [44], the Shiffman–Jarvik withdrawal scale score related to smoking items [45] and expired carbon monoxide (CO; ppm and %Hb). Principal component analysis was implemented to obtain a representative vector from these diverse measurements assessing smoking behaviours. The first principal component was used to calculate associations with brain features across participants for each repeated run. The behavioural data were missing from two participants; therefore, 29 participants were used for regression analysis. Potential outliers for the smoking measurements and brain features were considered based on median absolute deviation [60–62]. The statistical significance of the correlation coefficients was corrected using random permutations (*n* = 10 000), in which the regression analyses were conducted using randomly shuffled subject indices to generate a null distribution and consequently to obtain a corrected *p*‐value [11,62,63]. In addition, the 95% confidence interval for the correlation coefficients was obtained from 10 000 cycles of bootstrapping with replacement [56,62]. The *Q* test statistic for the test of heterogeneity was computed to compare the three correlation coefficients across the three repeated runs [64].

## 3. Results

### (a) Classification between repeated runs

**Figure 2.**
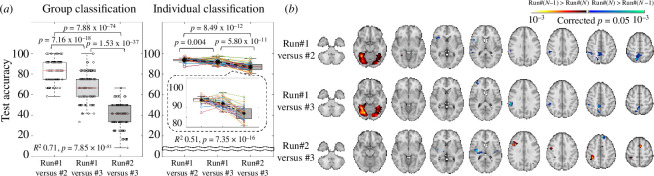
Performance for group and individual classification to discriminate whole-brain patterns between repeated runs combining ‘crave’ and ‘don’t crave’ conditions. (*a*) Test accuracy for each of group and individual classification, the *p*-values from two-sample (used for group classification) or a paired (used for individual classification) *t*‐test and linear regression across three contrast cases for each of group and individual classification were Bonferroni corrected by dividing it by the total number of samples used. (*b*) Identified brain regions via group inference using individual weight spatial patterns from each contrast case, Bonferroni-corrected *p* values < 0.05 from 10 000 random permutations.

Figure 2 shows the results of classification comparing pairs of repeated NF runs. The highest accuracy (83.79% ± 9.38%) for group-level classification was found in the comparison between the first run and second run (67.56% ± 12.91% and 39.01% ± 11.28%, run#1 versus run#3 and run#2 versus run#3, respectively). For individual-level classification, the accuracy of the first run versus the second run (93.78% ± 1.75%) was comparable to that of the first run versus the third run (92.12% ± 2.44%). Those accuracies were significantly higher than that of the second run and the third run (87.02% ± 3.38%; detailed information in figure 2*a*). The spatial patterns via group inference using individual weight features are illustrated in figure 2*b*. The fusiform area was identified from the classification of the first run versus the second run or the third run, and the precuneus, middle cingulate cortex, inferior parietal lobule, caudate and thalamus were also present from the same contrasts. From the comparison of the second and third runs, the ACC, angular gyrus, inferior parietal lobule and superior frontal gyrus were identified.

### (b) Classification between ‘crave’ and ‘don’t crave’ conditions

**Figure 3.**
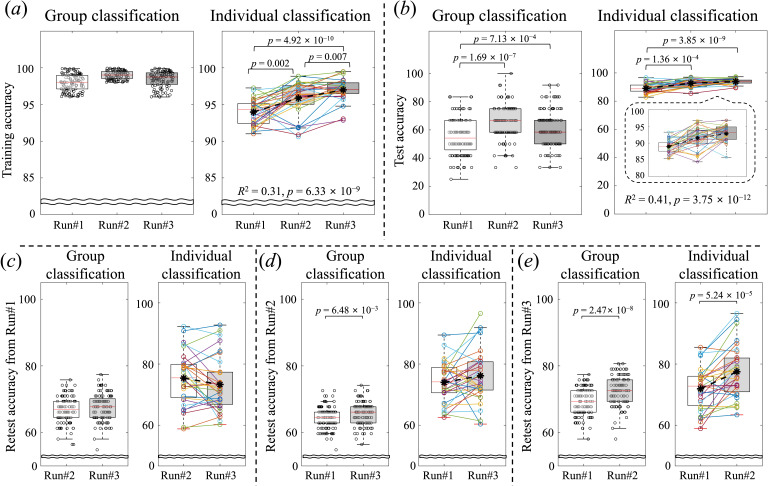
Performance for group and individual classification to discriminate ‘crave’ and ‘don’t crave’ conditions per each repeated run. (*a*) Training accuracy for each group and individual classification, (*b*) Test accuracy for each group and individual classification, (*c*) Retest accuracy using identified classifier of the first run to other runs, (*d*) Retest accuracy using identified classifier of the second run to other runs, (*e*) Retest accuracy using identified classifier of the third run to other runs. The *p*-values from two-sample (used for group classification) and a paired (used for individual classification) *t*‐test were Bonferroni corrected by dividing by the total number of samples used.

In figure 3, the group and individual classification accuracies are illustrated by comparing two conditions for each of the training, test and retest accuracies. Overall, it is notable that increased accuracy was evident from the individual classification across the repeated runs. More consistently increased performance in the individual classification across the repeated runs was observed for training and test accuracies compared to that of the group classification (figure 3*a,b*). From the retest analysis, the individual classification accuracies reported higher performance than the group classification accuracies and only when using a classifier from the last run showed significantly increasing performance for both group and individual classifications (figure 3*c*–*e*). The estimated motion (i.e. FD) was not significantly different between the ‘crave’ and ‘don’t crave’ conditions (*p* = 0.352), and motion parameters were not significantly associated with individual classification accuracy (detailed in electronic supplementary material, figure S2).

Comparing online and offline analyses, classification of ‘crave’ and ‘don’t crave’ conditions using c-SVM (used for online analysis) resulted in similar accuracies and trends as classification using ν-SVM (used for offline analysis). The group classification accuracy from c-SVM was lower than that from ν-SVM and showed no statistical significance between the first run and following runs. The results of individual classification from c-SVM showed statistical significance for the first and following runs and higher accuracy than that from ν-SVM (detailed in electronic supplementary material, figure S3).

**Figure 4.**
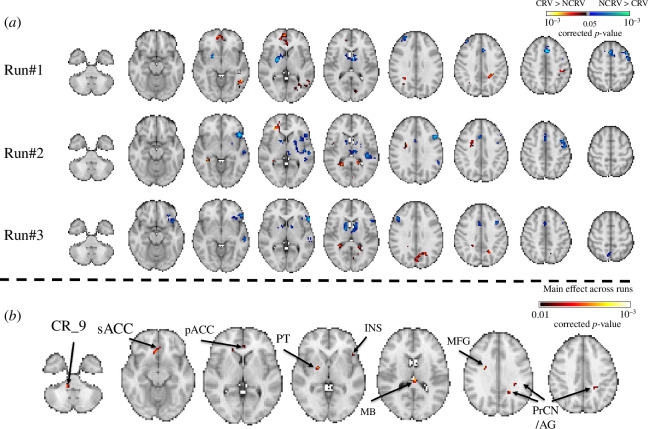
Identified brain regions via group inference in the case of classification between ‘crave’ and ‘don’t crave’ conditions. (*a*) Result of one-sample *t*‐test using individual weight spatial patterns for each run, Bonferroni-corrected *p* values < 0.05 from 10 000 random permutations. (*b*) Result of ANOVA test across the three runs, Bonferroni-corrected *p* values < 0.01 from 10 000 random permutations. CRV, crave; NCVR, don’t crave; CR, cerebellum; PT, putamen; INS, insula; MB, midbrain; MFG, middle frontal gyrus; PrCN, precuneus; AG, angular gyrus.

Figure 4 shows the results of classification to distinguish between the ‘crave’ and ‘don’t crave’ conditions using one-sample *t*‐test (figure 4*a*) and ANOVA (figure 4*b*). The visual, parietal and frontal areas showed statistical significance from overall *t*-tests of group inference, but the left anterior insula and bilateral caudate predicted the conditions from the following runs compared to the first run without feedback and the caudate and frontal areas showed decreased tendency across the repeated runs. Between the second and third runs, overall spatial patterns of the insula and frontal areas showed shrinkage patterns. The perigenual and subgenual anterior cingulate cortex (pACC and sACC), putamen, insula, midbrain, cerebellum, middle frontal gyrus, precuneus and angular gyrus were reported in the contrast of comparison across the repeated runs.

### (c) Relationship between identified brain regions and smoking measurements

The identified brain regions using ANOVA were further used to estimate a relationship between brain features (i.e. weight values) and smoking-related measures. The putamen and insula showed an increasingly positive correlation across repeated runs; the pACC, middle frontal gyrus, precuneus with angular gyrus and cerebellum represented more negative correlation through repeated runs and the ACC, sACC and midbrain represented mixed tendency of correlation across repeated runs. Among them, the insula (*Q* = 4.80, *p* = 0.045) and middle frontal gyrus (*Q* = 4.62, *p* = 0.049) showed marginal significance from the test statistic of correlation change across the NF runs (detailed in electronic supplementary material, figure S5).

## 4. Discussion

In this work, we have demonstrated the use of a machine learning-based rtfMRI-NF procedure with an updated classifier across repeated runs to assess modulating brain features associated with smoking craving. In line with traditional rtfMRI-NF based on activity or connectivity, our SVM-based rtfMRI-NF method appeared to modulate brain features in the regions associated with smoking craving. While group-based classification showed moderate accuracy for distinguishing ‘crave’ and ‘don’t crave’ conditions, the performance of individual classification was consistently improved across repeated NF runs with greater levels of accuracy compared with those obtained using group classification. Furthermore, the classification to discriminate between NF runs when collapsing ‘crave’ and ‘don’t crave’ or within condition represented comparable accuracy from the following runs compared to the first run in the individual classification but not in the group classification. These results suggest that the NF signal from a machine learning-based individually estimated model on the whole brain promotes the modulation of the BOLD signal in relevant brain regions associated with smoking craving.

### (a) Classification of repeated neurofeedback runs

The reported performance from group classification of the first run versus the second run had the highest accuracy when compared to the first run versus the third run and the second run versus the third run. Repeated NF runs may individually optimize functional processes to regulate brain and cognitive functions [42], such that individual brain regions related to regulation may be optimized and localized through repeated NF runs. In addition, the results from individual classification could provide evidence of the individual optimizing process, as shown by the comparable accuracies between the two contrasts of the first versus second runs and of the first versus third runs and through less variability in classification accuracy from the first versus third runs compared to that from the first versus second runs. The lowest accuracy produced when comparing the second run versus the third run can be explained by the fusiform area dominantly affecting the classification of the first, and subsequent, runs. Nevertheless, the brain regions associated with smoking craving and real-time NF learning were clearly identified as part of the central executive network (i.e. dorsal attention network or frontoparietal network) including the inferior parietal lobule, angular gyrus, dorsal lateral prefrontal cortex, ventral premotor cortex and middle temporal gyrus [65–67] and the default mode network including the precuneus [65,66]. Furthermore, the classification to compare two of the three runs by fixing ‘crave’ or ‘don’t crave’ conditions showed the same tendency, and of interest, the performance was higher from contrasts within the ‘don’t crave’ condition (94.48%, 94.91% and 90.79% for the case of run#1 versus run#2, run#1 versus run#3 and run#2 versus run#3, respectively) than those within the ‘crave’ condition (94.65%, 94.09% and 87.30%; detailed in electronic supplementary material, figure S4). While the effect of motion may be a contributor in some classifier-based paradigms [34,68], our findings indicate that motion parameters are not significantly related to individual classification. However, further work would need to consider motion differences between conditions or sessions in classifier-based real-time studies.

### (b) Classification of ‘crave’ and ‘don’t crave’ conditions

The performance from the group classification of ‘crave’ and ‘don’t crave’ conditions significantly improved in the runs following the initial run. However, the tendency of performance was not consistently improved across the repeated NF runs, whereas the individual classification showed increasing accuracy of the repeated NF runs significantly and steadily. In this study, we informed participants whether to ‘crave’ or ‘don’t crave’ but participants could choose their own strategies to regulate their brain functional process. Successful learning could vary, therefore, depending on the type of instructions given to participants or the unique strategies used by each participant [8,19,38], and the results of this study may indicate optimized or distinct learning processes for each individual arising across the repeated runs. It would be interesting to examine learning performance depending on individually chosen strategies for regulating their brain processes. That greater performance was also found in the retest classification at the individual level compared to the group level provides additional backing for this conclusion.

We identified brain regions via group inference using weighted spatial features across participants. Compared to the first run, the parietal areas, ventral and dorsal frontal areas and the insula showed shrinkage tendencies across the repeated NF runs. Meanwhile, the ANOVA comparing across the repeated runs revealed the right putamen, left insula, right middle frontal gyrus, left precuneus, left angular gyrus, right cerebellum, pACC and sACC, and midbrain. The frontoparietal network covering the middle frontal gyrus and angular gyrus in this study may have a functional role in regulating brain features during cognitive, affective and addiction-related processing [69,70]. In previous rtfMRI-NF studies of smokers, the activity level in the ACC and precuneus was positively correlated with subjective craving ratings [11,20,22]. These regions were also identified in our study despite our focus on the NF signals on the whole brain rather than information within ROIs. Additional identified brain regions included the cerebellum and midbrain, which have been previously reported to be related to smoking craving in the substantia nigra [71] and the central grey substance of midbrain [72]. Moreover, subcortical areas such as the putamen and insula could be implicated in optimizing the modulation of smoking craving, as a regression analysis with significant statistics of heterogeneity showed a relationship between brain features and smoking measures positively increasing across repeated NF trainings [65,73,74] (detailed in electronic supplementary material, figure S5).

### (c) Classification of group versus individual classification

This study compared classification performance at the individual (i.e. within-subject) and group (i.e. across-subjects) levels. Of note, we found that individual classification outperformed group classification for all contrasts, even though whole-brain multi-voxel pattern analysis faces potential issues such as the complexity of dimensionality, validation of optimal parameters and feature selection [75]. The degree of robustness and reliability of classification are of interest to many research areas, and previous studies have addressed the within-subject or across-subject variability affecting classification performance [76,77], namely that the within-subject consistency may not reflect across-subject reliability. The rtfMRI-NF techniques have commonly involved providing NF information to each participant with individually extracted brain features. Therefore, as evidenced by our findings, it may be possible that rtfMRI-NF contributes to increasing across-subject variability and optimizing within-subject functional processes. Further research could examine participants receiving NF information generated from group-level or individual-level classifiers.

### (d) Potential limitations and further work

It is important to note the limitations of our study. First, while comparable to other studies applying rtfMRI-NF methods to nicotine use disorder, the sample size (*n* = 31) was relatively small [11,20–22]. Additionally, the number of repeated runs and visits was limited, which may have restricted learning effects across repeated visits and runs. Real-world craving external to the lab environment was also not obtained [4,78]. Therefore, further investigation is warranted to evaluate the proposed methods in a larger study population, with the use of additional smoking behaviour/craving measures and as part of potential smoking cessation protocols. Further, individual craving responses to specific smoking images can vary across individuals and this variability can influence attention to smoking urges, potentially affecting the learning of NF regulation [79,80]. This potential effect could be included to explain across-subject and within-subject variability in future work. Finally, due to the limited number of samples for individual-level classification, we adopted different quantifications of neural activity for individual-level and between group-level classification, that is, a percentage BOLD signal was used for individual-level classification instead of beta values employed for group-level classification. Further work exploring the impact of different quantifications of neural information [81,82] should be considered when evaluating classifier-based performance in the NF field.

The reported rtfMRI-NF data obtained from online (i.e. application of c-SVM in electronic supplementary material, figure S3) and offline processing (i.e. application of ν-SVM in figure 2) showed a significant increase across the repeated runs of individual classification. The classification performance might be variable depending on the adopted model and its hyperparameters. Overall accuracy using c-SVM showed higher performance compared to that using ν-SVM; however, the consistently increased accuracy was found solely in the case of an individual classification of ν-SVM. Therefore, further work may consider potential issues regarding model variability and overfitting/underestimation [83,84].

It is important to note that brain regions can be selectively chosen for NF, such as the mesolimbic pathway including the ventral/dorsal striatum, nucleus accumbens, amygdala and anterior insula for reward-related systems [15,85], or the attention networks including the parietal cortex and dorsolateral frontal cortex for voluntary deployment of attention [15,86]. While this study used whole-brain features to classify NF runs or conditions, the identified brain regions were consistent with brain networks associated with reward processing and attention. Therefore, it would be interesting for future work to compare NF information within a network or across networks. Beyond the application of rtfMRI-NF for modulating smoking craving, a number of studies have also explored potential therapeutic applications for attention deficit hyperactivity disorder [87], chronic pain [5], major depression [88], Parkinson’s disease [89] and schizophrenia [10]. The present data suggest that whole-brain-based NF classifiers may facilitate modulation of brain function for neuropsychiatric disorders, particularly for modulating mental functions for which substantial indifferences may exist.

## 5. Conclusion

In this study, we have presented evidence supporting the feasibility of implementing NF signals via whole-brain machine learning-based individually optimized classifiers in the rtfMRI-NF method to distinguish craving and not craving in smokers. This approach has promising implications for personalized interventions, allowing individuals to regulate individualized brain patterns and associated mental functions. While further research is needed to explore generalizability, the present data represent a crucial step toward using rtfMRI-NF as a therapeutic tool in functional reorganization and personalized mental health treatment.

## Data Availability

Data underlying this article are made accessible through the Virginia Tech Data Repository [90]. Supplementary material is available online [91].
